# Maternal vitamin D deficiency associated with neonatal hypocalcaemic convulsions

**DOI:** 10.1186/1475-2891-6-23

**Published:** 2007-09-19

**Authors:** Laxmi Camadoo, Rebecca Tibbott, Fernando Isaza

**Affiliations:** 1 Senior Registrar in Paediatrics, Nambour hospital, Mapleton Road, Nambour, Queensland, 4560, Australia; 2 ST1 in Paediatrics, Ealing Hospital, Uxbridge Rd, Southall, UB1 3EU, London, UK; 3 Consultant Paediatrician, Northwick Park Hospital, Harrow, Middlesex, HA1 3UJ, UK

## Abstract

Maternal vitamin D insufficiency is not uncommon. Infants born to mothers who are deficient in vitamin D and or calcium, usually due to cultural modifications in their diets or clothing habits, and in addition are breastfed, are at risk of developing vitamin D deficiency and hypocalcaemia. We present a case of neonatal hypocalcaemic seizures secondary to vitamin D deficiency.

Rickets in children resulting from vitamin D deficiency is well documented. It is also becoming clear that there is a positive correlation between maternal vitamin D status during pregnancy and lactation and the development of rickets both in infancy and childhood. The correlation between maternal vitamin D, neonatal vitamin D and hypocalcaemia is not well documented.

## Background

We present a case of neonatal seizures secondary to hypocalcaemia where the only other abnormal findings were low vitamin D levels both in the infant and in the mother, and hypocalcaemia in the baby.

## Case Presentation

A one week old, full term male infant presented to Accident and Emergency with generalised seizures. He was exclusively breast fed since birth. Both parents were vegetarians, from Asian origin, and mother dressed in her cultural customs, where most of her body was covered. Mother was neither taking nutritional, nor vitamin supplements during pregnancy.

Physical as well as neurological examinations were within normal limits. His parents had recorded the "attacks" by video camera, and tonic-clonic generalised movements were well identified on the screen. Magnetic resonance imaging of the brain was normal. Electroencephalography showed no epileptiform phenomenon.

Laboratory investigations in blood, revealed calcium 1.58 nmol/L (6.33 mg/dl), 25-hydroxyvitamin D 37 nmol/L (2.69 mg/ml) indicating vitamin D deficiency and hypocalcaemia. His parathyroid hormone levels (PTH) was 6.8 pmol/L. Maternal 25-hydroxyvitamin D3 was 11 nmol/L (4.23 mg/ml); and her PTH was 18.6 pmol/L.

The normal levels in our laboratory are between 0.6 and 5.7 pmol/L for PTH, between 2.15 and 2.58 mmol/L for calcium. Vitamin D deficiency is defined as <12.5 nmol/L, and insufficiency levels between 12.5 and 50 nmol/L.

All other laboratory results (liver function tests, urea and electrolytes, C reactive protein, lumbar puncture, blood culture, lactate) were normal.

Baby did not receive anti-epileptic medications.

Within 6 hours of admission, once the initial laboratory tests became available, the patient was commenced on alphacalcidol (100 ng/kg once a day, which were reduced to 50 ng/kg/d after 1 week), and calcium supplements (0.25 mmol/kg/d).

After four days the calcium levels had returned to normal. He remained on alphacalcidol until ten weeks of age and then was changed onto Abidec 0.6 mls/d (vitamin A, B, C and D supplement). His seizures ceased within three days of starting treatment.

After 6 months, the baby was doing well, with normal calcium 2.42 mmol/L (9.7 mg/dl) and total 25 hydroxyvitamin D levels of 132 nmol/L (51 mg/ml), and his development was according to his chronological age.

## Discussion

It is well recognised that maternal vitamin D deficiency during pregnancy and during the period of breastfeeding contributes to the development of rickets in infancy [1-3]. However, neonatal hypocalcaemic seizures as a consequence of maternal vitamin D deficiency is not well described in western societies.

It is not uncommon to find vitamin D insufficiency in otherwise, healthy pregnant Women [4]. Infants born to such mothers have reduced umbilical cord blood concentrations of 25-hydroxycholecalciferol [5]. In addition, breast milk contains only about 1 microgram of vitamin D per litre. This varies according to maternal vitamin D status [6].

In 1991, the Committee on Medical Aspects of Food Policy recommended that all pregnant and lactating mothers should receive 10 micrograms vitamin D (400 IU) daily [7]. This policy has not been implemented widely.

Our case illustrates the importance of checking the calcium levels in neonates who present with seizures and in those found to be hypocalcaemic to check their vitamin D status as this is an easily correctable condition.

It could be argued that this case was also preventable had the mother been given vitamin D supplementation during her pregnancy and during her early lactation. However, given the lack of previous reports in the literature it would appear that neonatal hypocalcaemic seizures is a rare but real presentation of vitamin D deficiency.

## Competing interests

The authors declare that they have no competing interests.
